# High levels of childhood trauma associated with changes in hippocampal functional activity and connectivity in young adults during novelty salience

**DOI:** 10.1007/s00406-023-01564-3

**Published:** 2023-02-04

**Authors:** Mélodie Derome, Sandra Machon, Holly Barker, Petya Kozhuharova, Natasza Orlov, Elenor Morgenroth, Kenneth Hugdahl, Paul Allen

**Affiliations:** 1 Department of Psychology, School of Psychology, University of Roehampton, Whitelands College, Hollybourne Avenue, London, SW15 4JD UK; 2 Univ Lille, INSERM U1172, CHU Lille, Lille Neuroscience & Cognition Centre (LiNC), Plasticity & SubjectivitY Team, Lille, FR France; 3 Combined Universities Brain Imaging Centre, London, UK; 4 Department of Psychosis Studies, Institute of Psychiatry, Psychology & Neuroscience, King’s College London, London, UK; 5 Ecole Polytechnique Fédérale de Lausanne, Lausanne, CH Switzerland; 6 Department of Biological and Medical Psychology, University of Bergen, Bergen, Norway; 7 Division of Psychiatry, Haukeland University Hospital, Bergen, Norway; 8 Department of Radiology, Haukeland University Hospital, Bergen, Norway

**Keywords:** Schizophrenia spectrum, Neuroimaging, Development

## Abstract

Childhood trauma (CT) has been linked to increased risk for psychosis. Moreover, CT has been linked to psychosis phenotypes such as impaired cognitive and sensory functions involved in the detection of novel sensory stimuli. Our objective was to investigate if CT was associated with changes in hippocampal and superior temporal gyrus functional activation and connectivity during a novelty detection task. Fifty-eight young adults were assigned to High-CT (*n* = 28) and Low-CT (*n* = 24) groups based on their scores on the childhood trauma questionnaire (CTQ) and underwent functional Magnetic Resonance Imaging during an auditory oddball task (AOT). Relative to the Low CT group, High CT participants showed reduced functional activation in the left hippocampus during the unpredictable tone condition of the AOT. Furthermore, in the High CT group, psychophysiological interaction analysis revealed hypoconnectivity between the hippocampus and temporal and medial regions. The present study indicates both altered hippocampal activation and hippocampal-temporal-prefrontal connectivity during novelty detection in individuals that experienced CT, similarly to that reported in psychosis risk populations. Early stressful experiences and environments may alter hippocampal function during salient events, mediating the relationship between childhood trauma and psychosis risk.

**Supplementary Information:**

The online version contains supplementary material available at 10.1007/s00406-023-01564-3.

## Introduction

Childhood trauma (CT) is a common experience worldwide, and it is estimated about a third of the general population may be affected [1]. Adverse childhood events have been widely linked to an increased risk for psychiatric disorder and mental ill health (e.g. [2, 3]). In particular, there is growing evidence that CT can increase the risk of psychosis and psychosis like experiences [4–10], and may be a greater risk factor than a genetic predisposition, which is now thought to explain approximately 2% of the phenotypic variance in schizophrenia cohorts (Psychosis Endophenotypes International Consortium, 2014).

A seminal meta-analysis by Varese and colleagues [9] reported that the association between childhood adversity and psychosis was highly significant and that the estimated population attributable risk was 33% across studies. These findings indicate that childhood adversity is strongly associated with increased risk for psychosis in adulthood.

Further, the psychopathological outcomes associated with CT may be mediated by alterations in cognitive processes, that are also observable in the development of psychotic disorders [11]. In particular, CT has been linked to adverse cognitive consequences such as deficits in attention, memory, emotion regulation, and inhibitory functioning [12].

One cognitive process of interest when studying CT in relation to the development of psychosis, is novelty salience, which is thought to require sensory specific attention and memory as well as working memory processes involved in updating internal representations of the environment [13]. Previous studies demonstrate that these processes are altered in patients with schizophrenia and psychosis risk cohorts. A useful paradigm to investigate updating of working memory and cognitive control is to assess the neural systems associated with processing target stimuli in the context of oddball tasks [14]. Typically, in an oddball detection task, the target stimulus is presented much less frequently than the background, standard or regular stimuli, and successful task performance requires contextual attention and updating and working memory [15]. Patients with schizophrenia show diffuse hypo functioning during novelty processing in frontal, temporal and parietal cortices, as well as amygdala and thalamus [14]. In addition, recent studies suggested that neural response in the hippocampal-striatal-midbrain circuit during salience processing is altered in patients with positive psychotic symptoms [16, 17], and in patients in the early stages of psychosis development [18, 19].

Whilst psychosis [20–22] and psychosis risk states [23] have been linked to a range of functional, anatomical and neurochemical changes in the brain, recently a number of neuroimaging studies have also investigated the effects of childhood trauma on brain function and structure (e.g. [24, 25] for reviews). Intriguingly, there appears to be considerable overlap between neural changes seen in psychosis, and changes seen in an adult population that have experienced CT [26], regardless of whether a formal diagnosis is present or not [24]. One of the most robust neuroimaging findings in psychosis and schizophrenia populations is alterations in temporal lobe activation. Reduced or altered medial and lateral temporal lobe volume, activation and connectivity have been widely reported in psychosis populations (see [27]). In particular, altered superior temporal gyrus (STG) structure, function and connectivity is seen in psychosis and schizophrenia populations [28] and linked to the experience of auditory verbal hallucinations [29–36]. Given the established association between childhood trauma and auditory verbal hallucinations in adulthood [8], it is interesting that adults who have experienced CT also show altered structure in language and speech sensory regions encompassing the STG [37].

Previous functional neuroimaging studies in psychosis high-risk cohorts also report altered medial temporal lobe activity, perfusion, and connectivity in the hippocampus and parahippocampus [29, 38–40]. Behaviourally, altered hippocampal functional activity and connectivity (as part of a wider hippocampal-midbrain-striatal network) may underlie aberrant salience processing [19, 40–42] as well as the formation of delusions [43]. Moreover, in relation to CT, the hippocampus is a particular region of interest because the effects of stress and trauma, are known to affect the hippocampus, via the hypothalamic–pituitary–adrenal (HPA) axis [4, 11]. The hippocampus, which is involved in learning and memory, is particularly sensitive to stress [44, 45], and has shown structural [46, 47] and functional [48, 49] changes linked to early stress exposure.

The main objective of the present study, given the link between CT and psychosis and psychosis like experiences, was to investigate if CT is associated with changes in hippocampal and STG functional activity and connectivity during novelty detection. We hypothesised that, relative to a low CT group, young adults with high levels of CT would show altered functional activity and connectivity in STG and hippocampal regions of interest (ROIs) during an auditory oddball task, particularly during the unpredictable tone condition. We also predicted that in a high CT group, altered functional activity and connectivity in these ROIs would be associated with CT levels and psychosis like experience.

## Methods

### Participants

Fifty-eight participants were recruited through Facebook groups on two sites: Roehampton and Royal Holloway Universities student groups. They were selected from 230 respondents who completed a Qualtrics (https://www.qualtrics.com) screening survey, using the Childhood Trauma Questionnaire (CTQ) to establish two groups: High CT group (> 40.5, *n* = 29), and a Low CT group (< 29.5, *n* = 29) based on the upper and lower quartiles of the sample distribution of the first 100 respondents. Exclusion criteria included: presence of contraindications for MRI scanning (i.e. presence of metal, pregnancy, etc.), current use of prescribed medication for neuropsychiatric disorders, or history of psychiatric disorders and current use of illicit substances. These criteria were assessed via a self-report pre-screening survey. Absence of psychiatric or neurological diagnosis was assessed with two questions in the screening survey: “have you ever been diagnosed with a psychiatric condition (e.g. ADHD, depression, anxiety, mood disorders)?” and “Have you ever been diagnosed with a neurological disorder or disease (e.g. epilepsy, stroke, head injury, seizures, brain tumours, brain surgery, Parkinson’s disease)?”.

Participants in the Low and High CT groups were matched for age, gender, estimated IQ, tobacco, cannabis, and alcohol use. A total of six participants were excluded for data analysis due to missing or unusable MRI and/or questionnaire data. Thus, the final sample included 52 participants, with 28 participants included in the High-CT group and 24 participants included in the Low-CT group (Table 1). Written informed consent was obtained from all participants under protocols approved by the Ethical committee of Roehampton University. They were reimbursed to the amount of £20 for their visit.

**Table 1:** Demographic information and clinical assessment variables for High and Low CTQ groups. Presented as Mean(sd)

*Variables*	*High CTQ*	*Low CTQ*	*Statistic (t)*	*p-value*	*Effect size (Cohen’s d)*
*n*	28	24			
Age	20.8 (1.83)	20.1(1.64)	1.45	0.155	0.402
Sex	Male:*n* = 6, Female:*n* = 22	Male:*n* = 7, Female = 17	*X*^*2*^ = 0.413	0.521	Odd ratio = 0.662
Ethnicity	White British:12, White (other):8, Mixed white-Asian:1, Indian:1, Bangladeshi:1, Asian background (other):2, Chinese:1, Other:2	White British:15, White (other):6, Mixed White-Asian:0, Indian:1, Bangladeshi:0, Asian background (other):0, Chinese:1, Other:1	*X*^2^ = 4.67	0.700	Cramer’s *V* = 0.300
Level of education	A level:*n* = 12, BSc:*n* = 15, MSc:*n* = 1	A level:*n* = 12, BSc:*n* = 11, MSc:*n* = 1	*X*^2^ = 0.310	0.857	Cramer’s *V* = 0.077
Years in Education	15.3(3.32)	15.1(3.39)	0.255	0.800	0.0710
Employment level	Part time:*n* = 3, Student:*n* = 25	Part time:*n* = 3, Student:*n* = 21	*X*^*2*^ = 0.040	0.841	Odd ratio = 0.840
Handedness	Right:*n* = 26, Left:*n* = 2	Right:n = 18, Left:*n* = 6	*X*^2^ = 3.17	0.075	Odd ratio = 4.33
Tobacco: Cigarettes per day	0.380 (1.51)	1.55 (3.85)	− 1.391	0.171	− 0.4234
Alcohol: Units of Alcohol per day	1.554 (1.95)	1.59 (1.65)	− 0.075	0.940	− 0.0209
Alcohol use	never:3, occasionally:17, moderate w-e:5, uncontrolled w-e:2, moderate daily:1, uncontrolled daily:0	never:2, occasionally:11, moderate w-e:4, uncontrolled w-e:3, moderate daily:3, uncontrolled daily:1	*X*^2^ = 3.51	0.622	Cramer’s *V* = 0.260
WRAT-R	75.2 (4.65)	76.6 (5.14)	− 1.01	0.318	− 0.280
Digit span	17.8 (4.13)	17.6 (5.00)	0.188	0.852	0.0532
Verbal fluency	44.1 (10.6)	43.3 (13.0)	0.238	0.813	0.0662
CEQ	2.04 (3.32)	3.17 (7.21)	− 0.744	0.460	− 0.207
BSI-total	64.6 (37.0)	23.8 (16.9)	4.97	< 0.001	1.38
BSI paranoid	1.18 (0.672)	0.773 (0.635)	2.561	0.014	0.730
BSI-psychoticism	1.32 (0.840)	0.836 (0.727)	2.148	0.037	0.612
DASS-Total score	8.893 (7.157)	6.694 (4.357)	1.31	0.196	0.364
CTQ-total	57.2(12.4)	26.9 (1.45)	11.8	< 0.001	3.30
CTQ-emotional abuse	14.3(3.89)	5.71 (0.955)	10.5	< 0.001	2.93
CTQ-physical abuse	9.71 (4.86)	5.00 (0.00)	4.75	< 0.001	1.32
CTQ-sexual abuse	7.75 (4.54)	5.00 (0.00)	2.96	0.005	0.824
CTQ-emotional neglect	14.8 (3.89)	6.00 (1.41)	10.4	< 0.001	2.90
CTQ-physical neglect	10.7 (3.61)	5.17 (0.381)	7.43	< 0.001	2.07

### Clinical and cognitive assessments

Participants’ recall of childhood trauma was measured using the *Childhood Trauma Questionnaire* (CTQ, [50]), and is divided in five subscales: emotional, physical, sexual abuse, emotional, and physical neglect.

The *Cannabis experience Questionnaire* (CEQ) [51] total score was used to match Low and High CT groups for cannabis consumption, to control for potential effect of cannabis consumption on main variables of interest. We also assessed *Alcohol consumption* in terms of alcohol units per day and *Tobacco consumption* as cigarettes per day.

The *Brief Symptom Inventory* (BSI) was used to measure self-reported clinically relevant psychological symptoms [52]. The positive symptom distress indices (paranoia and psychoticism) were used to assess psychosis like symptoms in our participants and as correlates of interest in our functional activity and connectivity MRI analyses.

The *Depression and Anxiety Stress Scale* (DASS) [53] total score was used in second-level fMRI analyses as a covariate of no interest to control for current levels of negative emotions/states.

*Intellectual functioning* was measured with a validated short version of the Wechsler abbreviated scale of intelligence (WASI II; [54] and working memory was assessed with the digit span backward task [55]. These measures were used to ensure that our High and Low CT groups were matched for estimated IQ and working memory function.

Descriptive statistics and statistical comparisons between High and Low CTQ groups on all demographic data and questionnaire’s variables are presented in Table 1 and supplementary table S1, see supplementary material for more details.

### Experimental design: Auditory Oddball Task (AOT)

The AOT used consists of a classic auditory oddball paradigm adapted for fMRI block design, with alternating ON (9 blocks) and OFF (9 blocks) periods. During ON blocks participants were presented with three conditions: Predictable (P), Unpredictable (UP), and Passive listening (PL). During the P condition, a deviant tone interrupted a regular tone at predictable intervals. In UP condition, the deviant tone interrupted the regular tone at random, unpredictable, intervals. During PL, only the regular tone was presented with no deviant. The regular tone was set at 1000 Hz and the deviant tone was set at 1500 Hz. Noise reduction headphones (Sensimetrics Ltd.) were used to minimise scanner background noise and all participants reported that tone stimuli during the AOT were clearly audible. The sound level was adjusted for participants individually, however, mean sound level that participants received in CT high and Low groups did not differ in terms of decibel (mean High CT = 94.8, Sd = 3.11; mean Low CT = 95.5, Sd = 2.98; *t*(50) = − 0.794, *p* = 0.431). See supplementary material for a detailed description of the block’s durations.

### Data acquisition and analyses

Structural and functional MRI images were acquired using a 3 T Siemens Magnetom TIM trio scanner. Full acquisition details can be found in supplementary material. FMRI data were processed and analysed with the Statistical Parametric Mapping 12 (SPM12; Welcome Department of Neuroscience, London, UK) software package. Functional images were realigned, and participants’ motion did not exceed 3 mm in any of the six directions. Each participant’s structural image was co-registered and segmented. Finally, normalisation was achieved to 1 mm^3^ Montreal Neurologic Institute (MNI) space and images were smoothed with a 8 mm at full width half maximum three-dimensional Gaussian Kernel.

First-level models were designed to investigate main-effects of interest (P tone blocks vs. UP tone blocks). At the first level, the P and UP tone conditions were entered into a Generalized Linear Model (GLM) and contrast images were estimated for each participant using the estimated GLM parameters. The six movement parameters were included as additional regressors of no interests in the design matrix at the first-level analysis. To examine the main effect of task (UP tone blocks vs. P tone blocks), a second-level random-effects one-sample t-test was specified in SPM12. To examine the main-effect of group, a second-level random-effects independent sample t-test was specified with a between group factor of High and Low CT groups for the condition of interest (UP tone blocks < or > P tone blocks). As High and Low CT groups were matched for age, gender, and IQ, these variables were not included in the second-level GLM as covariates of no-interest. To control for current affective states, total DASS scores (centred around overall mean) were included as a covariate of no-interest in the second-level GLM.

We first used an initial cluster defining threshold of *p* < 0.05 uncorrected before we enforced a peak voxel-wise height threshold of family wise error (FWE) correction at a threshold of *p* < 0.05. We used small volume correction (SVC) for two *a-priori*, 8 mm regions-of-interest (ROI) in the bilateral hippocampus [± 38, − 16, − 14; Modinos et al., 2020] and bilateral STG [± 57, -27, 6; Mathiak et al., 2002].

### PPI connectivity analysis

A psychophysiological analysis (PPI) was used to measure functional connectivity between the chosen seed region(s) and each voxel in the whole brain during each experimental condition (UP > P). For the PPI analysis, two participants were excluded because they did not show any activation in the defined ROIs, the final groups for PPI analysis therefore included 28 High CT (mean age = 20.8, sd = 1.83) and 22 Low CT (mean age = 20.0, sd = 1.70) participants.

Based on the group GLM result (see Results), a 6 mm region of interest was defined around the SVC coordinates of interest [− 38, − 18, − 14], representing the group-effect (Low CT > High CT) in the left hippocampus. The PPI term was estimated as the first eigenvariates of the extracted BOLD signal of the seed volume of interest (VOI) for each subject. Haemodynamic deconvolution was used on the extracted time-series, which were then multiplied by the psychological variable (demeaned time course of the task) and re-convolved with the HRF to obtain the PPI interaction term. Time-series were not corrected for any covariates, as potential confounds were factored into the previous stages of analyses.

The psychological, physiological and interaction terms were entered in a GLM for each subject with the interaction term as regressor of interest. Subsequently they were entered in an independent two-sample t-test to examine differences between High and Low CT groups. All statistical whole brain maps were thresholded at *p* < 0.001 (uncorrected) and *k* > 50. For voxels that survived this threshold at the peak level, a cluster-extant family-wise correction (FWE-c) for multiple comparison at *p* < 0.05 was applied.

### Behavioural task

Behavioural data from the AOT task were analysed to determine omission and commission error rates. Omission errors appear when participants miss a response to the standard 1000 Hz tone. A commission error occurs when participants erroneously respond to the target deviant tone (1500 Hz). Response time and percentages of correct and incorrect responses were calculated across all trial types and contrasted between High and Low CT groups using independent sample *t*-tests.

## Results

### Participant characteristics, sub-clinical data

Participant demographic and clinical data are shown in Table 1. Overall, Low and High CT groups were matched for Age, Sex, Ethnicity, level of education, WRAT estimated IQ, cannabis, alcohol, and tobacco use. By design the High CT groups had significantly higher CTQ scores (total and subscales) relative to the Low CT group. As would be expected, participants in the High CT group also had significantly higher BSI and DASS scores (total and subscales).

### Behavioural AOT performance

During the AOT High and Low CT groups did not differ in terms of task performance with both groups completing the task with a high level of accuracy: Overall, 94.18% (sd = 9.84) correct responses for the Low CT group and 91.69% (sd = 11.6) correct responses for the High CT group (*t* = 0.807, *p* = 0.423).

Error Rates: High and Low CT groups did not differ in terms of omission errors (failing to respond when presented with a stimulus tone of 1000 Hz). Error rates were 6.30% (sd = 12.2) in the Low CT group, and 8.40% (sd = 14.4) in the High CT group (*t* = − 0.547, *p* = 0.587; see Supplementary Figure S1.*C*). High and Low CT groups differed slightly in terms of commission errors (erroneously responding when being presented with the deviant tone of 1500 Hz). Error rates were 3.78% (sd = 4.34) in the Low CT Group and 7.98% (sd = 8.92) in the High CT group (*t* = − 2.049, *p* = 0.046; see Supplementary Fig. S1.*D*). Relative to the Low CT groups, the High CT group made more commission errors during the P tone blocks (Low CT, 2.09% (sd = 3.84); High CT, 6.84% (sd = 8.16); *t* = − 2.547, *p* = 0.014, see Supplementary Fig. S1.*F*). However, no group difference was observed during the UP tone blocks (*t* = − 1.182, *p* = 0.243, see Supplementary Fig. S1.*H*).

Reaction Times: Mean reaction times for correct trials (correctly pressing the button when presented with the stimulus tone of 1000 Hz), did not differ between groups (Low CT, rt = 0.430 s; High CT, rt = 0.428; *t* = 0.06, *p* = 0.946). See Supplementary Table S3 and Supplementary Figure S1.*I*.

### fMRI Analysis of AOT: Task Effects

**Fig. 1:**
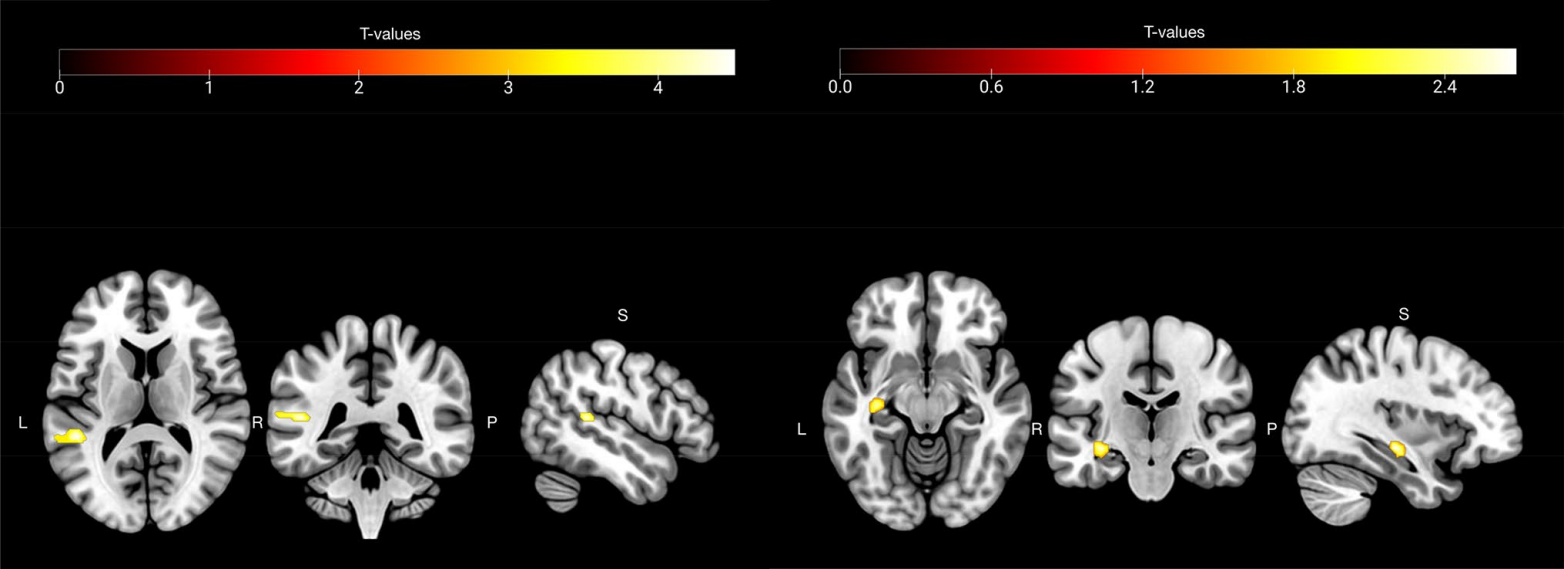
*Left Panel: Activation Maps showing Task effects*: the contrast unpredictable tone blocks > predictable tone block was associated with greater activation in the Left STG. *Right Panel: Activation Maps showing Group effects*: reduced activity in the left hippocampus in the High CTQ relative to the Low CTQ group during unpredictable > predictable tone blocks. All results are corrected using Family Wise Error thresholds *p* < .05

Relative to P tone blocks, during UP tone blocks there was greater activation in the STG ROI (left STG: *x* = − 54, *y* = − 32, *z* = 10, *Z*_peak_ = 2.51, *p(FWE)* = *0.048*). There were no regions within the STG ROI that showed greater activation during P tone blocks relative to UP tone blocks. Relative to P tone blocks, during UP tone blocks activation in the hippocampal ROI was observed at an uncorrected threshold but was non-significant after peak level correction was applied (*x* = − 32, *y* = − 16, *z* = 10, *Z*_peak_ = 1.67, *p(FWE)* = *0.16*). See Fig. 1 (Left side) and Table 2A).

**Fig. 2:**
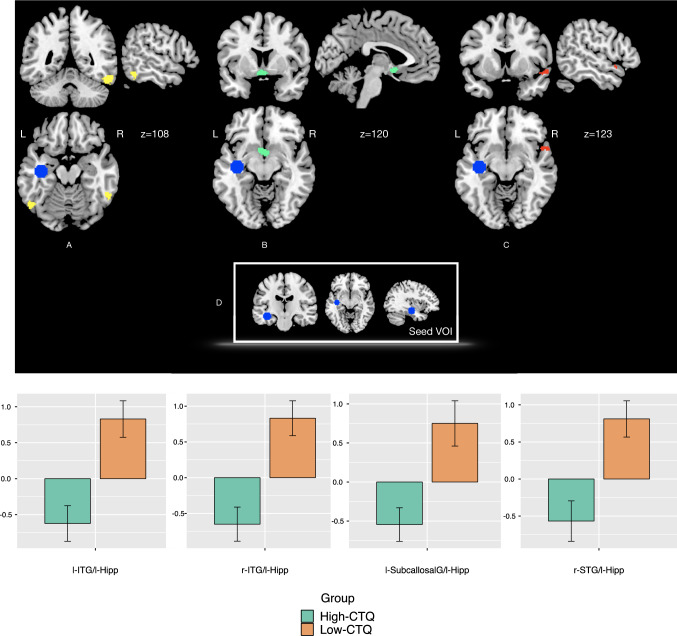
Functional connectivity (PPI) during UP > P tone blocks: regions exhibiting reduced functional connectivity with the seed VOI (Left hippocampus showed in dark Blue and in box D) in the High CTQ group. **A** Right and left inferior temporal gyri (ITG) in Yellow; **B** left subcallosal gyrus (l-SubcallosalG) in cyan; **C** right superior temporal gyrus (r-STG) in red. *p*(FWE) < 0.05. Bar charts illustrate reduced functional connectivity (PPI parameters) in the High-CTQ group relative to the Low-CTQ group

**Table 2:** MNI coordinates and labels of areas showing significant A) increased activation unpredictable > predictable contrast; B) decreased activation in unpredictable vs predictable contrast between groups (High CTQ < Low CTQ); C) Reduced functional connectivity (PPI parameter estimates) based on the left-hippocampus seed region (High CTQ group < Low CTQ group)

k	t	P (unc)	MNI(x,y,z)	Hemisphere	Lobe	Region	BA
A) Task Effect							
122	2.60	0.006	− 54, − 32, 10	L	Temporal	Superior Temporal Gyrus	41
	2.51	0.008	− 60, − 32, 10	L	Temporal	Superior Temporal Gyrus	22
	2.36	0.011	− 60, − 30, 6	L	Temporal	Superior Temporal Gyrus	42
	2.32	0.012	− 62, − 24, 8	L	Temporal	Superior Temporal Gyrus	42
B) Group Effect							
56	2.73	0.004	− 34, − 20, − 10	L		Parahippocampal gyrus, Hippocampus	
	2.44	0.009	− 34,− 18,− 14	L		Parahippocampal gyrus, Hippocampus	
	2.36	0.010	− 36,− 18,− 18	L	Temporal	Sub-Gyral	20
C) PPI analysis							
134	4.79	0.001	50, − 48, − 22	R	Temporal	Inferior Temporal Gyrus	37
73	4.58	0.012	− 54, − 64, − 18	L	Temporal	Inferior Temporal Gyrus	37
92	4.01	0.006	− 4, 6, − 10	L	Frontal	Subcallosal Gyrus	25
51	3.84	0.031	58, 10, -8	R	Temporal	Superior Temporal Gyrus	38

*Note*. Coordinates of peak effects are provided in MNI space, Montreal Neurological Institute, BA, Brodman area. Neuroanatomical locations of activations were identified using Talairach Client software

### fMRI analysis of the AOT: group effects

In the STG ROI there were no significant group effects for the contrast UP tone > P tone blocks. In the hippocampal ROI during the UP tone > P tone block contrast, the High CT group showed reduced activation in the left hippocampus (*x* = − 34, *y* = − 20, *z* = − 10, *Z*_peak_ = 2.62, *p(FWE)* = 0.036) relative to the Low CT group (see Fig. 1 (Right panel), Table 2B)). There were no areas in the right hippocampal ROI where activity was greater in the High relative to the Low CT group.

### Functional connectivity: PPI effects during unpredictable versus predictable condition

The between-group comparison of PPI (functional connectivity) effects during UP tone > P tone blocks showed that compared to the Low CT group, the high CT group had reduced functional connectivity between the left-hippocampus (seed region) the bilateral inferior temporal gyri (right (*x* = 50, *y* = − 48, *z* = − 22): Z_peak_ = 4.53, *t* = 4.79, *p(FWE)* = 0.001; left (*x* = − 54, *y* = − 64, *z* = − 18): Z_peak_ = *4.09, t* = 4.28, *p(FWE)* = 0.012); left subcallosal gyrus (x = − 4, *y* = 6, *z* = − 10; Z_peak_ = 3.84, *t* = 4.01, *p (FWE)* = 0.006); and right superior temporal gyrus (x = 58, y = 10, z = -8; Z_peak_ = 3.69, *t* = 3.84, *p (FWE)* = 0.031). See Table 2C) and Fig. 2.

## Discussion

We examined the functional architecture of two regions of interest, i.e. the hippocampus, and superior temporal gyrus (STG), during a novelty salience task in young adults reporting exposure to childhood trauma (CT). We chose to focus on these regions as functional activation and connectivity in the hippocampus and STG have previously been shown to be altered in schizophrenia [14, 16, 17] and early psychosis cohorts [18, 19, 40] during salience and oddball tasks.

Although the effect of conditions in the hippocampal region of interest failed to reach a corrected level of statistical significance, possibly due to adaptive habituation [56, 57], we did observe a group-effect in hippocampal functional activation and connectivity. In line with our first prediction, individuals with higher levels of CT (High CT group) showed reduced hippocampal activation during the unpredictable tone condition, suggesting dysregulation at the neural substrate of salience processing in this group. We then used a seed-based approach to examine functional coupling between hippocampus and the whole brain during salience processing. Findings revealed that the High CT group showed significantly reduced functional connectivity between the left hippocampus (seed region) and inferior and superior temporal gyri, and the medial PFC during the unpredictable tone condition. Previous findings have shown the same left-hemisphere bias in victims of abuse, with reduced connectivity notably in typical attention network [58]. Moreover, in the context of novelty detection of faces, studies showed altered activation of the left hippocampus related to degree of exposure to childhood maltreatment [59–61]. Thus, whilst using a different salience paradigm to those used in previous studies (Blackford, Allen, Cowan, & Avery, 2013; Edmiston & Blackford, 2013; Hart et al., 2017), we showed similar CT-related alterations in the hippocampus during a novelty salience/detection task. We also add to previous findings by showing that during salience detection, CT is associated with reduced hippocampal-lateral temporal-mediofrontal functional connectivity.

During the unpredictable (UP) tone condition (relative to the predictable (P) tone condition), we did observe increased activation in the left STG (a task effect). Increased activation in the STG region is thought to correspond to the evoked P300 component (a component sensitive to unpredictable events) and appears to involve a distributed network including, temporo-occipital and superior temporal regions [62–65]. Furthermore, Downar and colleagues [66], suggested that the STG plays a central general role in identifying salient stimuli within the sensory environment across modalities. Contrary to our prediction however, we did not observe a group effect for functional activation in the STG region of interest during the AOT. However, functional connectivity between the hippocampal seed region and inferior and superior temporal gyri was reduced in the High CT group relative to Low CT group. Previous ERP work [67, 68] suggests that the presentation of deviant tones is associated with bidirectional connectivity changes within temporal and frontal regions which may be important for inferring the level of predictability of sensory inputs. Our findings suggest that, although no differences in functional activation were seen between Low and High CT groups, wider functional networks, that include lateral temporal lobe hubs, are affected by CT.

We also predicted that these functional changes in the High CT group would be associated with psychosis like experiences. Indeed, in the High CT group, changes in left hippocampus activation and connectivity during the unpredictable tones condition were similar to those reported in schizophrenia [14, 16, 17] and clinical high-risk groups (Allen, Chaddock, et al., 2012; Allen et al., 2011; Modinos et al., 2020; Winton-Brown et al., 2017), congruent with the notion that altered hippocampal activation during stimulus novelty are present in psychosis risk cohorts. However, in the present study we did not test for a statistical association between reduced hippocampal activation and connectivity and psychosis like experiences. Thus, it remains unclear if changes in hippocampal function due to CT increases the likelihood of psychosis like symptoms or increases risk for the psychotic like experiences.

The psychophysiological (PPI) analysis revealed reduced functional connectivity in the High CT group during novelty detection, between the hippocampus and bilateral inferior temporal gyri, right STG, and middle prefrontal cortex. This novelty detection network includes sensory cortices, medial PFC, and the anterior hippocampus, regions that have been identified in several studies using various experimental novelty-detection paradigms [69–72]. Interestingly, our findings are in line with existing literature, showing that alterations in the hippocampus were associated with exposure to stress, trauma and childhood maltreatment [73, 74]. Overall, the present results seem to point towards alterations in a network implicated in novelty detection, including hippocampal, temporal, and mid PFC regions, that are associated with more physical and sexual forms of childhood maltreatment.

### Limitations

Although we had an adequate sample of participants to detect functional group effects [75], these results would benefit from replication in a larger sample. Our definition of high and low CT groups was also somewhat arbitrary and based on the upper and lower quartiles of the 100 first respondents. As such, results must be interpreted with caution. Furthermore, the CTQ is a self-report retrospective measure which relies upon autobiographical recall that may be biassed by current affective states [76]. Besides, we could not assess whether the traumatic events in the High CT group were suffered during early childhood or more recently in mid adolescence, this should be taken into consideration when investigating further brain function and development. Also, we classified our participants based on the total CTQ scores, however abuse profiles might differ in terms of structural, behavioural and psychiatric consequences [77, 78]. Thus, different profiles (sexual abuse, physical abuse, emotional abuse) might be associated with different neural signatures. Furthermore, we did not detect hippocampal activation at a corrected threshold level for the main task effect, during unpredictable tones relative to predictable tones. Whilst our a priori ROI was sensibly informed by findings from a previous study ([40]), an ROI approach may have limited observable effects in the present study in two ways. First, there may be extrahippocampal tissue or CSF included within the spherical ROI used, contributing to noise in the BOLD signal. Second, childhood trauma is associated with reductions in hippocampal volume. It may be the case that more extrahippocampal tissue or CSF is being included in the high CT group as a result. Finally, the hippocampus can be divided in subfields, which might be differentially affected by childhood trauma. Thus, replication of the results with inclusion of hippocampal subfields might bring new and more precise insight on the specificities of this region related to aberrant salience processing and childhood trauma.

## Conclusions

The findings from the present study indicate both altered hippocampal activation and hippocampal-temporal-prefrontal connectivity in the context of novelty salience in individuals who reported previous childhood trauma. Whilst these functional changes appear to be linked to childhood maltreatment, the association with psychosis like experiences, and potentially psychosis risk, was not established. Nevertheless, the neural signature of novelty salience processing/detection in a high CT group is similar to that seen in psychosis and psychosis risk cohorts. Future neuroimaging research need to focus on how childhood trauma influences the stress-response system which in turns may affect medial temporal function, and consequently the function of brain areas important for cognition such as novelty salience sustained by the hippocampus and prefrontal regions [44].

## Supplementary Information

Below is the link to the electronic supplementary material.

Supplementary file1 (DOCX 268 KB)

## Data Availability

Data can be made available upon request.
