# Supplementary material for: High levels of childhood trauma associated with changes in hippocampal functional activity and connectivity in young adults during novelty salience
Source: Eur Arch Psychiatry Clin Neurosci. 2023 Feb 4;273(5):1061–72. doi: 10.1007/s00406-023-01564-3 (PMC10359215; doi:10.1007/s00406-023-01564-3)
Supplement: Supplementary file 1 — Supplementary file1 (DOCX 268 KB) [file 406_2023_1564_MOESM1_ESM.docx]

**Supplementary Material**

**Supplementary Table S1**.

| *Variables* | *High CTQ* | *Low CTQ* | *Statistic (t)* | *p-value* | *Effect size (Cohen’s d)* |
| --- | --- | --- | --- | --- | --- |
| BSI-somatisation | 1.21(0.733) | 0.669(0.644) | 2.727 | 0.009 | 0.777 |
| BSI-obsessive compulsive | 0.911(0.715) | 0.492(0.557) | 2.256 | 0.029 | 0.643 |
| BSI-interpersonal | 0.714(1.14) | 0.466(0.769) | 1.124 | 0.267 | 0.320 |
| BSI-depression | 1.14(0.783) | 0.583(0.574) | 2.809 | 0.007 | 0.800 |
| BSI-anxiety | 1.06(0.720) | 0.621(0.704) | 2.157 | 0.036 | 0.615 |
| BSI-hostility | 1.29(0.979) | 0.800(0.756) | 1.919 | 0.061 | 0.547 |
| BSI-phobic anxiety | 1.26(0.909) | 0.773(0.757) | 0.040 | 0.047 | 0.581 |
| DASS-depression | 12.0(9.43) | 3.79(4.06) | 3.98 | <0.001 | 1.11 |
| DASS-stress | 13.8(7.89) | 7.25(5.43) | 3.42 | 0.001 | 0.951 |
| DASS-anxiety | 8.96(7.54) | 3.33(3.25) | 3.40 | 0.001 | 0.945 |
| Resilience factor (CD-RISC) | 61.750(18.368) | 69.750(8.872) | -1.95 | 0.057 | -0.541 |

Clinical assessment variables subscales for High and Low CTQ groups.

**Supplementary Table S2**.

Beta values of between-regions connectivity in the High-CT group when compared to the Low-CT group.

| *Regions* | *Mean (sd) Sample (n=50)* | *Mean(sd) High CTQ (n=28)* | *Mean(sd) Low CTQ (n=22)* | *Statistics* | *p* |
| --- | --- | --- | --- | --- | --- |
| R-Inferior Temporal Gyrus/L-hipp | 0.00163(1.41) | -0.650(1.26) | 0.831(1.15) | -4.29 | <0.001 |
| L-Inferior Temporal Gyrus/L-hipp | 0.0160(1.44) | -0.623(1.31) | 0.829(1.19) | -4.05 | <0.001 |
| L-Subcallosal Gyrus/L-hipp | 0.0251(1.39) | -0.544(1.13) | 0.750(1.36) | -3.67 | <0.001 |
| R-Superior Temporal Gyrus/L-hipp | 0.0390(1.48) | -0.567(1.44) | 0.810(1.15) | -3.66 | <0.001 |

*Note*. Values are presented as Mean(sd).

**Supplementary Table S3**.

Behavioural analysis of the AOT, between groups (High CT vs Low CT) statistics are presented.

| *Variables* | *Mean (sd) Low CTQ* | *Mean(sd) High CTQ* | *Statistics (t)* | *p* |
| --- | --- | --- | --- | --- |
| Nb correct | 228.870 (23.906) | 222.808(28.111) | 0.8074 | 0.423 |
| Nb errors | 14.130(23.906) | 20.192(28.111) | -0.8074 | 0.423 |
| Nb errors (P) | 2.609(4.2398) | 4.962(5.7095) | -1.6197 | 0.112 |
| Nb correct (P) | 78.391(4.2398) | 76.038(5.7095) | 1.6197 | 0.112 |
| Nb errors (UP) | 3.174(4.3448) | 5.885(7.7062) | -1.4894 | 0.143 |
| Nb correct (UP) | 77.826(4.3448) | 75.115(7.7062) | 1.4894 | 0.143 |
| Nb omission errors | 11.913(23.0077) | 15.885(27.3062) | -0.5466 | 0.587 |
| Nb commission errors | 2.043(2.3448) | 4.308(4.8147) | -2.0489 | 0.046 |
| Nb omission errors (P) | 2.043(4.0393) | 3.154(4.4783) | -0.9066 | 0.369 |
| Nb commission errors (P) | 0.565(1.0369) | 1.846(2.2035) | -2.5474 | 0.014 |
| Nb omission errors (UP) | 1.522(3.0131) | 3.538(7.6275) | -1.1876 | 0.241 |
| Nb commission errors (UP) | 1.565(2.0632) | 2.462(3.0755) | -1.1815 | 0.243 |
| RT correct trials (stimulus tone) | 0.430(0.0839) | 0.428(0.0654) | 0.0683 | 0.946 |
| RT incorrect trials (deviant tone) | 0.379(0.1421) | 0.450(0.1422) | -1.5264 | 0.135 |

*Note:* Nb represent the number of correct or erroneous answer/trial. P represent the Predictable condition and UP the unpredictable condition (when discarding the rest of trial: rest period and passive listening). Omission errors: when participants should have pressed the button (stimulus tone of 1000Hz) but they did not. Commission errors: when participants should have ignored the deviant tone (1500Hz), but they pressed the button. RT correct trials represent the reaction time when participants responded correctly to the stimulus tone. RT incorrect trials represent the reaction time when participants answered to the deviant stimulus although they should not have.

**Supplementary Figure S1**.

Results of the behavioural analysis of the AOT task. The High CT group made significantly more commission errors, particularly in the predictable condition (P).

*Note*: * represent a statistically significant difference (p<0.05)

**Supplementary details regarding clinical and cognitive assessments**

The CTQ comprises 28 items designed to quantify self-reported childhood trauma history in the home. CTQ subscales scores present test-retest reliability coefficients ranging from 0.79 to 0.86, and internal consistency between 0.66 and 0.92 across the initial validation sample (Bernstein et al., 2003). Responses are measured on a 5-point Likert scale (from 1=never true to 5=very often true), each subscale consists of 5 questions with a score ranging from 5 to 25. In the overall sample of 230 respondents, scores ranged from 25 to 103.

The BSI consists of 53 items covering nine symptom dimensions: somatization, obsession-compulsion, interpersonal sensitivity, depression, anxiety, hostility, phobic anxiety, positive symptom distress index and positive symptoms total. The global indices measure current or past level of symptomatology, intensity of symptoms, and number of reported symptoms.

The DASS is a 42-items self-report instrument designed to measure current (state) levels of three related negative emotional states of depression, anxiety and tension/stress.

*Resilience* was measured with the Connor-Davidson Resilience Scale 25 items (CD-RISC-25, Connor & Davidson, 2003), including several components: self-efficacy, optimism, sense of humour, patience and faith in coping with stress and adversity.

**Supplementary information regarding the AOT blocks**

ON blocks were 30s long, and we used 27 trials with a ratio of 1:3 of deviant tone to regular tone in P and UP tone blocks (9 deviant, 18 regular). Trials were 1.1s long (0.1s for each tone, followed by 1s of silence). Participants were asked to press a response button when they heard the regular tone (1000Hz), and to withhold their response (not press the button) for the deviant tone (1500Hz). During OFF blocks, participants were told to rest and look at the fixation cross. UP, P and PL tone conditions were pseudorandomised (the 3 conditions were randomized across subjects, but all subjects performed the same blocks), and the task lasted for 10 min 35 seconds (4 min 30 sec for the task; 6 min rest; 5 sec buffer at the end).

**Supplementary details regarding data acquisition**

Structural and functional MRI images were acquired using a 3T Siemens Magnetom TIM Trio scanner equipped with a 32-channel head coil at the Combined Universities Brain Imaging Centre (CUBIC; http://www.cubic.rhul.ac.uk/). Structural images were acquired using weighted Magnetization Prepared Rapid Acquisition Gradient Echo (MPRAGE) T1 sequence with 176 slices and an in-plane resolution of 256 x 256, repetition time (TR) of 1900ms, 1mm x 1mm x 1mm voxel size, and scanning time of approximately 5 minutes. Functional images were acquired using an EPI sequence with 330 volumes, with a TR/TE/flip angle = 1000 ms/33 ms/70^o^, field of view 192 x 192 mm, slice thickness of 3 mm, giving a voxel size of 3mm x 3mm x 3mm, and a whole brain coverage of 48 interleaved slices.

**Supplementary Analysis 1: Correlation with clinical variables**

Regarding the correlation analysis between PPI parameters and clinical variables included in the manuscript, we additionally checked correlation with all clinical variables and subscales (such as those included in Supplementary Table S1).

In the High CT group, a positive correlation was observed between PPI connectivity between hippocampus/right inferior temporal gyrus and the BSI phobic anxiety subscale (*r*=0.401, *p*=0.035).
